# More than the Infinite Monkey Theorem: NHP Models in the Development of a Pediatric HIV Cure

**DOI:** 10.1007/s11904-023-00686-6

**Published:** 2024-01-16

**Authors:** Jairo A. Fonseca, Alexis C. King, Ann Chahroudi

**Affiliations:** 1 Department of Pediatrics, Emory University School of Medicine, Atlanta, GA USA; 2 Emory National Primate Research Center, Emory University, Atlanta, GA USA; 3 Emory+Children’s Center for Childhood Infections and Vaccines, Atlanta, GA USA

**Keywords:** HIV cure, Pediatric, Nonhuman primate, Reservoir

## Abstract

**Purpose of Review:**

An HIV cure that eliminates the viral reservoir or provides viral control without antiretroviral therapy (ART) is an urgent need in children as they face unique challenges, including lifelong ART adherence and the deleterious effects of chronic immune activation. This review highlights the importance of nonhuman primate (NHP) models in developing an HIV cure for children as these models recapitulate the viral pathogenesis and persistence.

**Recent Findings:**

Several cure approaches have been explored in infant NHPs, although knowledge gaps remain. Broadly neutralizing antibodies (bNAbs) show promise for controlling viremia and delaying viral rebound after ART interruption but face administration challenges. Adeno-associated virus (AAV) vectors hold the potential for sustained bNAb expression. Therapeutic vaccination induces immune responses against simian retroviruses but has yet to impact the viral reservoir. Combining immunotherapies with latency reversal agents (LRAs) that enhance viral antigen expression should be explored.

**Summary:**

Current and future cure approaches will require adaptation for the pediatric immune system and unique features of virus persistence, for which NHP models are fundamental to assess their efficacy.

## Introduction

According to UNAIDS, 1.5 million children 14 years or younger are living with HIV. This age group also represents 10% of the total incidence [1]. Pediatric infections often result from vertical transmission in utero, intrapartum, or during breastfeeding. HIV infection progresses faster in children than in adults, with higher peak and set-point viral loads. By the age of 2, mortality approaches 50% [2–4]. Antiretroviral therapy (ART) significantly reduces mortality [5, 6], but it does not eradicate the infection, leading to viral rebound upon ART interruption. The resulting lifelong treatment need brings several challenges, including adherence, ensuring consistent access, and comorbidities from ART adverse effects and chronic inflammation [7, 8].

The best characterized HIV cellular reservoir consists of latently infected memory CD4 + T cells carrying transcriptionally silent but replication-competent HIV [9–11]. Age-related differences in the composition and activation of the immune system and the viral dynamics of infection shape key reservoir characteristics. Therefore, it is essential to recognize that the advances in understanding viral persistence made in adults on antiretroviral therapy (ART) cannot be simply extrapolated to children and infants with HIV infection.

Children exhibit distinct patterns in the composition of their reservoirs, with a predominance of transitional memory CD4 + T cells [12]. This is different from adults, in whom central, transitional, and stem cell memory T cells represent the majority of the long-lasting HIV reservoirs [9, 13, 14]. Notably, our group has demonstrated that naïve CD4 + T cells are a significant component of the reservoir in infant nonhuman primates (NHPs) [15, 16]. Furthermore, intact HIV provirus has been identified in naïve CD4 + T cells from children living with HIV [17]. Beyond the differences in the reservoir composition, latently infected cells from individuals with perinatally-acquired infection appear significantly more resistant to reactivation than those from individuals with adulthood-acquired infection [18].

Understanding the children-specific differences in the formation and persistence of the reservoir and the impact of ART timing is crucial for developing a pediatric HIV cure. Infants with perinatal HIV, transmitted during late gestation or delivery, have the opportunity to start ART soon after birth (potentially even before diagnosis according to current treatment guidelines in North America and Europe), limiting the reservoir establishment and size and preserving the immune system function [19, 20]. Early ART is also associated with low levels of circulating viral antigen, potentially leading to decreased HIV-specific T and B cell responses [21–23]. However, infants who acquire HIV through breastfeeding or families lacking routine prenatal/postnatal care may face delays in diagnosis or ART initiation, resulting in different reservoir and immune characteristics compared to infants who start ART early.

NHP studies are crucial for the understanding of virus persistence and testing cure-directed strategies. Simian immunodeficiency virus (SIV) infection in Asian rhesus macaques reproduces the viral and immune features of HIV infection, including effective transmission through mucosal routes, gradual CD4 + T cell depletion leading to AIDS, and viremia that can be suppressed with ART [24]. NHP models allow the evaluation of cure strategies yet to be proven efficacious in vivo with an unknown safety profile and, therefore, with ethical limitations to justify their immediate testing in human study participants [25]. Moreover, these models allow a high degree of experimental control over the different infection variables, including virus dose, exposure route, time of ART initiation and interruption, and access to tissues that are not possible to obtain from human study participants, but that are of value as critical sites of viral replication and persistence [26, 27].

Unlike the infinite monkey theorem, which suggests that an infinite number of monkeys typing randomly will eventually produce a masterpiece [28], the use of NHP models as a means to reach a pediatric HIV cure is not about testing an infinite number of animals with an endless number of approaches. This review summarizes how the systematic study of retroviral infection of infant macaques has shaped our understanding of the interactions between the pathogen and the host during the immune system development and how these observations have led towards the formulation of potential pediatric HIV cure approaches. We also provide our perspective on novel methods to target reservoirs that should be pursued in infant rhesus macaques to provide rationale (or not) for clinical studies in children.

## Overview of the Pediatric Immune Response to HIV

The unique pathogenesis of HIV in children can be attributed to differences between the immune environments in early life versus adulthood. In early life, the immune system is in a dynamic discovery phase, in a constant balancing act between tolerance and inflammatory responses. Initially, the tolerogenic immune environment of the fetus prevails in the neonate, which prevents harmful pro-inflammatory responses during exposure to various neoantigens [29]. Resulting in decreased antiviral responses and rapid HIV progression [30] with higher peak viral loads and viral setpoints in infants compared to their adult counterparts [3, 4]. After infection, overwhelming immune activation leads to CD4 + T cell depletion, immune dysregulation, and disease progression [31]. We will briefly review the anti-HIV immune response in infancy and childhood but direct the reader to published comprehensive reviews [30–33].

### Innate Responses

In early life, the response to toll-like receptor (TLR) stimulation is distinct from that of adults, favoring the production of cytokines that promote the differentiation of T helper cells towards a Th2, Th17, and a regulatory phenotype [34, 35]. This differential response prevents the deleterious effects of uncontrolled inflammation against commensal antigens [36], and it is also protective against bacterial and fungal pathogens at the cost of impaired antiviral responses [31]. After perinatal HIV infection, both plasmacytoid and myeloid dendritic cells (DCs) become depleted, with those remaining having an impaired function, further reducing proinflammatory responses [37]. Importantly, ART in children can replenish the levels of myeloid DCs. Still, it only has a partial effect on the recovery of plasmacytoid DCs [37, 38], meaning that the production of type I interferons (IFNs) is reduced. Children with perinatal HIV also exhibit reduced NK cell cytolytic activity, including antibody-dependent cellular cytotoxicity (ADCC), which could contribute to rapid disease progression [39, 40]. However, in vitro models have shown neonatal NK cells can decrease HIV infectivity through the secretion of β-chemokines (CCL3, CCL4, and CCL5) which function as CCR5 ligands [41]. Garcia-Broncano et al. found that an NK cell activation phenotype, characterized by increased expression of the cytotoxicity marker CD57 and the decreased expression of the inhibitory marker NKG2A, was associated with a reduced viral reservoir in a cohort of early treated infants [42]. In adults, specific interactions between the protective HLA-B Bw4-80Ile allele and killer immunoglobulin receptors (KIR) are associated with both a delayed progression to AIDS and elite controller phenotypes mediated by improved cytotoxic responses [43–45]. Further studies are needed to define if there are protective or harmful MHC-KIR associations related to a progression phenotype in children.

### Adaptive Responses

Broadly neutralizing antibodies (bNAbs) develop during chronic HIV infection, and their generation is intricately linked to extensive somatic hypermutation and affinity maturation [46]. Children living with HIV develop bNAbs within the first 2 years of infection, while this process tends to take much longer in adults. Muenchhoff, et al. found that 70% of children developed bNAbs with a breadth of neutralization equivalent to that of the top 20% of bNAbs in adults [47]. Although the specific epitopes targeted by pediatric bNAbs often overlap with those in adults, they also exhibit remarkable diversity in terms of polyclonality, with some bNAbs capable of targeting up to four distinct epitopes [48]. It has been proposed that the high antigenic load seen in pediatric HIV infection enhances antibody breadth [49].

Unlike bNAbs, the production of antibodies with the ability to mediate ADCC is delayed in infants [50]. That said, passively acquired (through the placenta or breast milk) IgG1 antibodies with ADCC function are associated with improved survival in infants living with HIV [50]. In addition, ADCC capabilities are associated with non-progression in children [51]. Initiation of ART modulates humoral immune responses, given that viral suppression also reduces antigen exposure. A consistent feature of children who initiate ART early in the neonatal period is seroreversion, with loss of ELISA positivity [52].

The presence of Gag-specific CD4 + T responses has been associated with improved survival and lower viral loads in children [53]. CD4 + T cells also provide critical help for the generation of antiviral CD8 + T cells, and Sandberg et al. demonstrated that in neonatal HIV infection, there is a correlation between depleted CD4 + T cell levels and impaired anti-HIV CD8 + T cell responses [54]. Early ART initiation, which is feasible in newborns with ready healthcare access, significantly decreases the immune activation associated with HIV infection and is associated with improved T cell function in children [19, 55]. However, ART does not fully recover CD4 + T cells [56], and limiting antigen exposure through very early ART may, in fact reduce virus-specific adaptive immunity. It has also been observed that pediatric non-progressors have lower levels of immune exhaustion markers in both central and transitional memory CD4 + T cells and higher CD4 + T cell polyfunctionality when compared to those with typical disease progression [57, 58].

HIV-specific CD8 + T cell responses can be detected in the first week of life in neonates with in-utero HIV infection [59]. These cells are functionally impaired, with decreased IFN-γ production, compared to older children [60]. Furthermore, while in adults, the emergence of HIV-specific CD8 + T cell responses is correlated with a rapid but partial control of viremia in acute infection [61, 62], this rapid decline is not observed in children [63]. This effect is explained by the impaired CD8 + T cell responses. Different CD8+ T cell specificities can lead to different outcomes, increased polyfunctional Gag-specific CD8 + T cells are associated with slower disease progression and elite control in perinatal HIV infection; meanwhile, CD8 + T cells specific for Nef are associated with disease progression [58]. Protective HLA alleles able to mediate CD8 + T cell cytotoxicity through interaction with the T cell receptor have been described [64]. In the mother, protective HLA alleles can lead to the accumulation of viral immune escape mutations. In children with discordant HLA alleles compared to their mothers, slower disease progression is associated with effective immune responses [65]. However, for those children who inherit the maternal HLA alleles, uncontrolled viral replication can result from transmission of a founder virus that contains escape mutations [66]. Interestingly, adolescent elite controllers with perinatal HIV have a low prevalence of HLA alleles associated with viral control in adults, indicating that other factors mediate viral suppression in the pediatric population [32].

### Sex-Related Differences

Adland et al. observed in a South African cohort of women who acquired HIV during pregnancy that females were significantly more likely to become infected with HIV in utero than males, even though the proportion of males and females born to seroconverting women was not significantly different. The mechanism for this increased susceptibility appears to be related to higher levels of immune activation in females, permitting transmission of viruses resistant to type I IFN that also have reduced replicative capacity [67]. Males with perinatal HIV transmitted from mothers with recently acquired HIV also appear to have a greater ability to control viremia during periods of ART non-adherence [67, 68]. These findings merit further study as they suggest cure approaches may be more effective in males. Pediatric NHP models, if sufficiently powered to assess sex differences, may help to evaluate this hypothesis.

## Key Features of SIV/SHIV Infection and Persistence in Infants

### Oral Transmission as a Major Route of Infection

Without antiretroviral therapy, the risk for HIV transmission through breastfeeding is as high as 42% [69]. Further, HIV acquisition during delivery commonly occurs through the exposure of mucous membranes such as the mouth to virus in blood or vaginal secretions. Understanding the events leading to infection of the oral mucosa, subsequent viral dissemination, and the establishment of the latent reservoir are of particular importance for understanding HIV pathogenesis in children.

SIV and simian human immunodeficiency virus (SHIV) can also establish infection in infant rhesus macaques following oral inoculation [70–72]. Within 7 days from an oral challenge, systemic viral infection is established [73] and CD4 + T cell levels begin to decrease [74]. Studies that evaluate the role of breastmilk in promoting or restricting oral infection in infant NHPs have been informative. It has been observed that viral concentrations in the breastmilk of SIV-infected dams are similar to HIV levels in human breastmilk [75]. Both cell-free and cell-associated HIV levels in breast milk are significantly associated with transmission, with higher levels of either one associated with a higher risk of infection [76]. When compared to the rates for breast milk HIV transmission in humans, SIV transmission rates via breastmilk are higher in rhesus macaques [77] and lower in sooty mangabeys (natural hosts for SIV) [78]. Notably, this effect is independent of the viral load in the dam’s breast milk. Instead, it is associated with the levels of target cells for infection (CD4 + CCR5 + T cells), particularly in the oral and upper gastrointestinal tract tissues [79]. Breastmilk also contains innate immune factors with antiviral effects that may be protective against HIV/SIV transmission [80]. Using a SHIV model, with the SIV *env* gene replaced by an HIV *env*, Himes et al. tested the impact of a combination of anti-Env monoclonal antibodies (mAbs) isolated from lactating mothers living with HIV in the rates of infection of infant macaques. The mAbs were given both intravenously and mixed with the inoculum used for oral SHIV challenges. Although not statistically significant, the ADCC functionality of the mAbs was correlated with a decrease in the proportion of infants infected after oral SHIV challenge [81]. Similarly, the protective effect of antibodies with ADCC capabilities has been demonstrated in the sexual transmission of HIV in adults [82].

### Virus Dissemination Post Oral Challenge

**Fig. 1:**
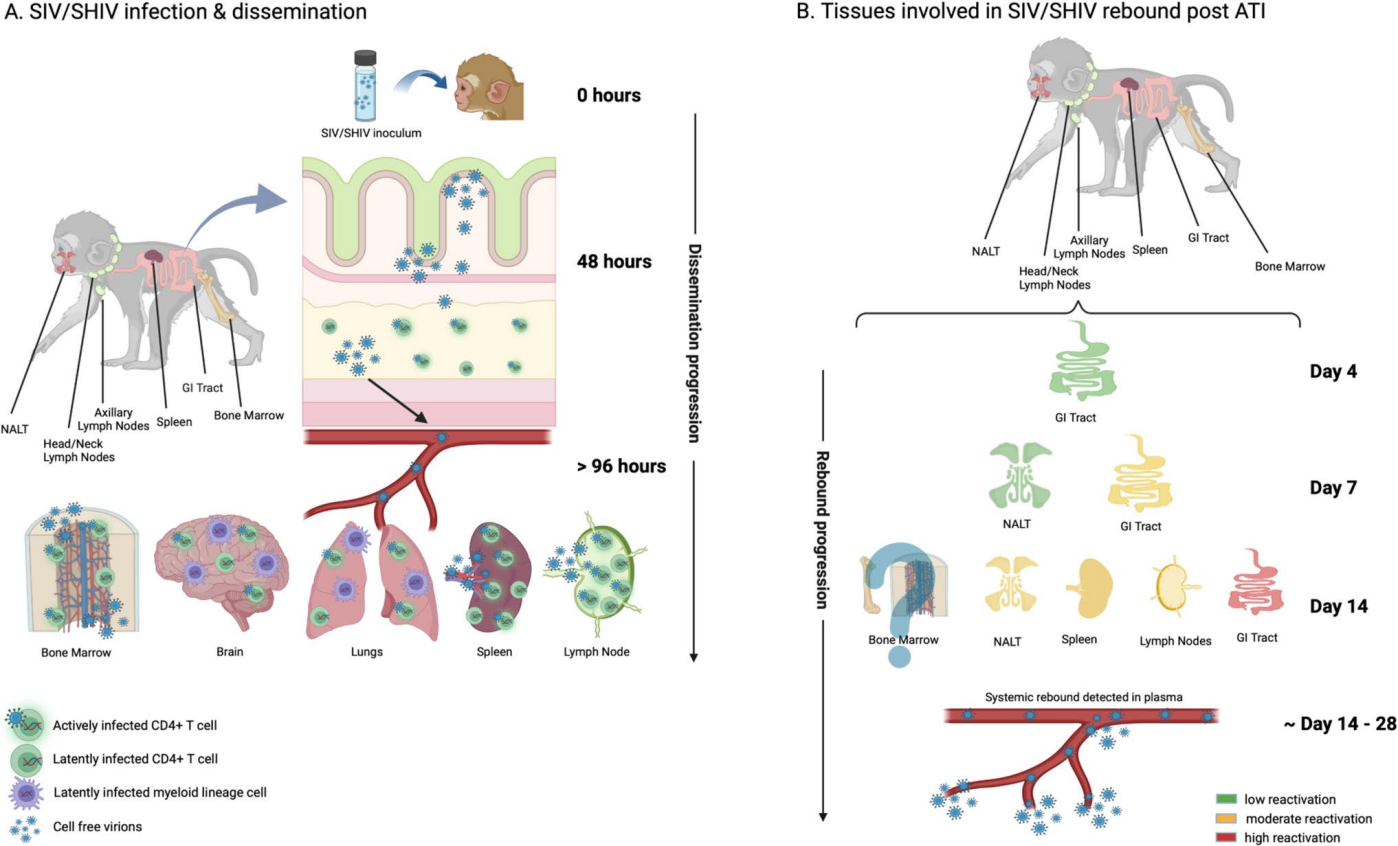
SIV/SHIV infection and rebound. **A** SIV/SHIV infection and dissemination after oral (p.o.) challenge in infant rhesus macaques. In the first 48 h, SIV/SHIV crosses the epithelial layer of the oral and upper gastrointestinal (GI) mucosa, infecting the gut and associated lymphoid tissues. After 96 h, SIV/SHIV traverses the lower intestinal mucosa and systemic infection takes place through hematogenous and lymphatic dissemination with establishment of active and latent infection in multiple tissues. **B** The timing of virus reactivation following analytical treatment interruption (ATI) in selected tissues is shown. Over the first 2 weeks of ART interruption, increases in viral expression are seen first in the GI tract, followed by nasal-associated lymphoid tissue (NALT), spleen and lymph nodes. Bone marrow also represents a potential source of virus reactivation. The increase in viral replication in tissues is then detectable in the plasma as viremia ~ 14–28 days after ART interruption. Created with BioRender.com

Once SIV infection is established, the virus rapidly disseminates to lymphoid and non-lymphoid tissues (Fig. 1A). Within 1 day of oral SIV_mac251_ challenge, the virus is detected in the oral mucosa and esophagus, along with lymph nodes in the head, neck, and axillae of neonatal rhesus macaques [70]. By 72 to 96 h, the infection spreads to the intestinal and colonic mucosa, mesenteric lymph nodes, and as far as the lungs and the brain [70, 75, 83]. Using SHIV_1157ipd3N4_ for oral challenge, the palatine tonsils were found to be a key site of infectious virus, although this was measured 8 weeks into infection [81]. In terms of the specific cell types involved in early virus dissemination, Taylor et al. showed that 96 h after oral inoculation with SHIV_1157idp3N4_, most infected cells were T cells located in both the small and large intestine [84]. In SIV models, both macrophages and CD4 + T cells are positive for viral RNA and DNA in mucosal and lymphoid sites post-oral infection [70, 83]. Notably, compared to adults, neonatal macaques have higher levels of activated and proliferating CD4 + T cells, particularly in the intestinal mucosa, which are more susceptible to SIV infection and depletion [85]. In fact, when clinical circumstances have permitted the study of gastrointestinal tract tissues from human feti and neonates (autopsies and biopsies), activated CD4 + T cells expressing the HIV/SIV coreceptor CCR5 are readily identified, including Th17 cells [86]. In contrast, CD4 + T cells in cord or neonatal blood have low CCR5 expression. Th17 cells play a vital role in maintaining the integrity of the intestinal mucosal barrier [87]. In adults, HIV infection leads to a preferential depletion of Th17 cells, which compromises gut integrity, facilitates microbial translocation, and triggers immune activation [88–90] that, in turn, facilitates HIV replication, CD4 + T cell depletion, and disease progression [30]. Similar data have not been generated following perinatal infection. However, HIV exposure has been shown to disrupt the Th17:Treg balance in the blood of infants [91], and loss of Tregs from intestinal tissues of neonatal macaques following SIV infection has been described [92]. It is likely that Th17 cells are depleted in the GI tract of infants upon HIV/SIV infection as well. These findings, combined with the observation that the upper oral mucosa is an early target in oral infection, highlight the importance of the gastrointestinal tract in early viral replication and dissemination. However, other concomitant events are likely involved in this process, as shown by Milush et al., where a role for systemic dissemination via the lymphatic system starting from infection of the tonsils and lymph nodes in the head and neck was observed [70].

### Phenotypes of Disease Progression

After HIV infection is established in children, the progression to AIDS is faster compared to adults. This can be partly explained by a still-developing immune system with a bias towards T helper 2 (Th2), Th17, and regulatory T cell (Treg) responses [35, 93]. These responses are considered to have evolved to prevent proinflammatory Th1 responses in utero and as newborns are exposed to novel environmental antigens [29]. This tolerogenic environment limits the induction of antiviral cellular immune responses, including against HIV, thus contributing to the persistently higher viral loads seen in infants and children beyond the acute phase of infection as compared to adults [30]. However, high viral loads alone do not explain pediatric HIV disease progression, as approximately 10% of ART-naïve children have normal CD4 + T cell levels in the setting of uncontrolled viremia [63]. It has been suggested that a combination of persistent immune activation, the exhaustion of CD8 + T cells [49], and the failure to develop neutralizing and cytotoxic T cell responses is associated with rapid progression [51, 94].

The association between a lack of antiviral antibody responses and rapid progression to AIDS has also been observed in SIV-infected rhesus macaques [95]. Wood et al. [96] orally inoculated infant rhesus macaques and divided the infants into rapid progressors (defined as those with high viral loads with undetectable or very low levels of specific SIV-env antibodies), and typical progressors. In this study, 44% of the infected rhesus macaques (11/25) showed a rapid progressor phenotype. The authors’ found that the rapid progressive phenotype was associated with the activation of plasmacytoid dendritic cells in lymph node germinal centers, leading to elevated levels of type-I interferon (IFN) in B cell follicles. These high type-I IFN levels are associated with the depletion of follicular T helper (TFH) cells and a failure to expand memory B cells, ultimately impairing the production of SIV-specific antibodies [96].

SIV models are also able to provide insight into pediatric HIV non-progressors. SIV infection of natural host species, such as sooty mangabeys, results in high levels of viral replication with maintenance of healthy peripheral CD4 + T cell counts, similar to pediatric non-progressors. Reduced immune activation in the chronic phase of infection can explain the decreased immunopathology in sooty mangabeys, leading to lower levels of CCR5 expression and decreasing the targets for infection and viral replication. In particular, central memory CD4 + T cells express lower levels of CCR5 upon activation compared to effector memory CD4 + T cells, resulting in a preserved central memory pool [97, 98]. Muenchhoff et al. showed that ART-naïve pediatric non-progressors have similar findings to those observed in SIV infections of natural hosts with reduced CCR5 expression and lower cell-associated HIV levels in central memory CD4 + T cells [47]. Furthermore, they demonstrated low levels of immune activation, consistent with the observation that aberrant persistent immune activation after HIV infection is one of the main drivers of pediatric progression. There are some differences in the immunologic phenotypes of natural hosts and pediatric non-progressors (e.g., magnitude/breadth of CD4 + T cell responses [99]), and a thorough understanding of the full range of mechanisms involved in protection from disease may inform the development of therapeutics based on these observations. While certainly not a cure in the traditional sense, the lessons provided by natural hosts teach us about the host–pathogen interactions that contribute to immunity and avoidance of chronic inflammation.

### Anatomic Distribution of the Latent Reservoir

Although ART has significantly increased the life expectancy of children and effectively controls virus replication, the formation of the latent reservoir composed of infected cells carrying a transcriptionally silent virus that remains replication-competent makes a cure with ART alone virtually impossible [100]. Early ART initiation in the first days to weeks of infection can restrict reservoir size (i.e., the number of infected cells that contribute to viral recrudescence if ART is stopped) [101], and there is evidence that early ART also decreases aberrant immune activation and induces improved antiviral responses [19]. However, the efficacy of early ART could also depend on virological and host factors [102]. Moreover, several case reports show a prolonged period of remission with no detectable viremia after stopping ART in patients who received early treatment [103–105]. Nonetheless, there is no clear understanding of the mechanisms involved in the virus control after analytical treatment interruption (ATI) of ART, and biomarkers predictive of HIV rebound in children are only recently emerging [106].

NHP models allow researchers to circumvent several of the challenges of studying human infants, as well as permitting the control of variables related to infection and treatment, providing a greater understanding of the events that lead to reservoir formation and latency reactivation. Our group and others have developed both SIV [15] and SHIV [16, 107] models of oral transmission and ART suppression with similar viral and CD4 + T cell kinetics to those observed in pediatric HIV infection before and after treatment. Using multiple methods to study virus persistence in tissues of infant macaques, we and others have demonstrated the presence of viral DNA in lymph nodes, spleen, gastrointestinal (GI) tract, liver, and the brain during the suppression of viremia on ART [15, 16, 107, 108]. Infection of myeloid lineage cells in the lungs has also been described during acute SIV infection of infant macaques [109], although data on ART are scant for this age group.

In our SIV model [15], using RNAscope and DNAscope in situ hybridization, we observed that the SIV DNA levels were significantly lower in both the lymph nodes and the spleen of ART-suppressed infants when compared to viremic ART-naïve infant macaques, but no difference was seen between ART-treated infants and ART-treated adults. This pattern was also observed in the gut lymphoid aggregates. Some tissues, including the lamina propria of the GI tract, showed SIV DNA levels that were similar in treated and untreated animals. RNA levels were significantly lower in all lymphoid tissues as well as in the gut lamina propria in the setting of ART-suppressed viremia. Interestingly, in infants infected with SHIV.C.CH505 [16], levels of SHIV RNA in CD4 + T cells isolated from rectal biopsies remained stable between 8 and 48 weeks of ART while declining in both peripheral blood and lymph node CD4 + T cells. Moreover, the SHIV RNA:DNA ratios were 1 log higher in the GI tract than those in the blood and lymph nodes, indicating persistent transcriptional activity while on ART. Persistent viral transcription in the rectum has also been observed in ART treated, SIV-infected adult macaques, albeit at a lower frequency when compared to untreated animals [110]. It is unclear if lower ART tissue concentrations, an increased frequency of target cells [79], or intrinsic characteristics of the immune system in the gut are responsible for the persistent transcription despite ART.

Several studies have shown SIV dissemination into the brain of infant macaques between 24- to 72-h post-challenge [111]. We found low levels of SIV DNA and RNA in the brains of both untreated and ART-suppressed infants; however, differences in viral levels were not seen based on treatment status. This finding appears consistent with low or undetectable concentrations of the ART drugs tenofovir, emtricitabine, and dolutegravir in the brain [15]. Given observations that SIV preferentially localizes in myeloid cells in the brain, our group evaluated the cells associated with viral nucleic acid by DNA/RNAscope in situ hybridization and immunohistochemistry, finding only CD163 + (myeloid) and not CD3 + (lymphoid) cells to contain SIV RNA in the brain of infant macaques [15]. Upon further study, when CD11b + myeloid cells were isolated from the brains of ART-treated SHIV.C.CH505-infected infant rhesus macaques, 2/6 (33%) animals had low levels of viral DNA and 1/6 (17%) showed viral RNA expression [16]. Understanding the factors that define susceptibility to infection and viral persistence in the central nervous system (CNS) is critical, as it has been observed that infectious, replication-competent virus can be isolated from CNS macrophages/microglia [112, 113] and that the CSF is an independent source of viral RNA production in adults living with HIV after ART interruption [114, 115]. Furthermore, future cure strategies must be evaluated for their efficacy in myeloid cells.

### Cellular Distribution of the Latent Reservoir

While attention to myeloid cells is important, it must be acknowledged that in the peripheral blood, lymph nodes, and other lymphoid sites (spleen, Peyer’s patches of the GI tract), the reservoir is mainly comprised of CD4 + T cells. Defining the involvement of specific CD4 + T cell subsets and any age-related differences that exist is a critical step towards further elucidation of the mechanisms of reservoir establishment and maintenance, as well as to assess the need for subset-specific cure strategies. CD4 + T cells can be subdivided according to helper cell polarization, functional properties, and tissue localization. Our group evaluated the contribution of the naïve, stem cell (SCM), effector (EM), transitional (TM), and central memory (CM) CD4 + T cells to the total SIV or SHIV viral reservoir in ART-treated infant macaques. SIV/SHIV DNA was detected in all evaluated T cell subtypes in both adult and infant macaques. In adults, TM and CM CD4 + T cells contributed the most to the SIV reservoir [15]. In contrast, naïve CD4 + T cells were the main contributors to the total CD4 + T cell SIV and SHIV reservoir in infants, as this subset is the largest CD4 + T cell population in infants and both intact and replication-competent virus was isolated from these cells [15, 16]. These observations are consistent with the reports of replication-competent HIV isolated from naïve T cells in adults living with HIV [116, 117]. Elucidating the effect naïve cell differentiation into memory cells and their subsequent expansion has in the reservoir persistence, as well as how their resistance to immune clearance [118] could affect immune approaches to a pediatric HIV cure, is currently a focus of our research.

During SHIV infection, infants have significantly higher proportions of lymph node TFH cells when compared to adults. However, there were no differences in SHIV-specific TFH between the groups [119]. The frequency of TFH cells and associated germinal centers declines after about 2 weeks following SIV infection of infant macaques relative to uninfected infants [120]. As resident TFH cells in both the tonsils and the gastrointestinal-associated lymphoid tissues (GALT) are capable of supporting viral replication post ATI in SHIV-infected infant macaques [107], TFH are likely infected and depleted during active viral replication. Our group has demonstrated the presence of SIV DNA in TFH cells from mesenteric lymph nodes but did not find differences in viral DNA levels between these cells and other T cell subsets after SIV suppression by ART (initiated at 4 weeks of infection) in infants [15]. We did observe a significantly increased frequency of SIV DNA in TFH cells compared to other T cell subtypes in superficial lymph nodes. These observations make it necessary to evaluate not only the contribution of the TFH cells to the reservoir, but also the effects that the timing of ART initiation may have on reservoir distribution and composition.

### Sources of Viremia Post-ATI and Associated Immune Determinants

The duration of infection prior to ART start, as well as the duration of ART, have been shown to influence viral rebound kinetics in adult rhesus macaques [121–123]. To assess the influence of early ART on viral rebound in the pediatric setting, Wang et al. challenged newborn rhesus macaques with SIVmac251 intravenously 6 h after birth, started ART 3-day post-infection, and then stopped ART 9 months later [124]. Four out of 5 animals had no evidence of viral rebound, and the animal that rebounded was the only one with evidence of integrated SIV DNA in axillary lymph nodes and detectable levels of SIV RNA in the rectum. Our group has performed a study of staggered ART start after oral SHIV.C.CH505 infection in 4-week-old infant macaques, finding that 25/30 animals rebounded when ART was stopped after 1 year of treatment [125]. Of the five non-rebounding animals, four were in the earliest ART start group (day 4–7 after infection). While differences in virus, route of infection, and timing/duration of ART make comparisons between these studies challenging, both support the use of early ART in infants to restrict reservoir establishment and potentially influence post-ART viral control, with case reports of children with perinatal HIV infection underscoring the impact of early ART [103–105].

In most cases, however, if treatment with ART is interrupted, either due to non-adherence or as part of a study protocol, viral rebound typically follows within a few weeks. The re-establishment of HIV viremia after ART is discontinued is likely multifocal, with multiple anatomic and cellular reservoirs contributing to the viral rebound (Fig. 1B) [126, 127]. Our group assessed viral rebound kinetics and origin during ATI in SHIV.C.CH505-infected infant rhesus macaques [128]. These animals started treatment 8-week post-infection and were maintained on ART for 52 weeks. Viral rebound occurred 7 to 35 days after ATI, and viral loads reached pre-ART levels in most cases. To further characterize the anatomic sources of rebound, a radiolabeled gp120 V2 apex-binding PGT145 monoclonal antibody was used to detect viral expression using ImmunoPET scans. These scans were performed once on long-term ART and then twice weekly from the start of ATI. The nasal-associated lymphoid tissue, axillary lymph nodes, spleen, and the GI tract were the sites with substantial PET signal, even before viremia was detectable. The earliest and greatest signal of virus reactivation occurred in the GI tract, consistent with our observations of active viral transcription in the rectum despite ART-mediated suppression of viremia [16]. Goswami et al. analyzed the levels of cell-associated infectious SHIV in tissues obtained 8 weeks post ATI after a short 8-week course of ART in infant macaques [107]. Post-ATI, the infectious virus was mainly distributed in the gut and lymph nodes. Interestingly, there was a higher proportion of animals with infectious viruses in the submandibular versus the mesenteric lymph nodes, again highlighting the relevance of examining the oral-associated lymph nodes in terms of viral persistence in addition to infection and dissemination [70, 83].

Elucidating the role of the immune system in the dynamics of rebound viremia is critical not only to develop new immunotherapeutics aimed towards viral control off ART but also to identify biomarkers that predict the risk of viral progression for clinical studies evaluating the efficacy of cure-directed interventions. In the SHIV infection model, animals expressing the Mamu-A*01 MHC I allele had prolonged periods of post-treatment control and lower viremia after ATI, showing the importance of cytolytic T cell (CTL) responses [128]. CTL responses associated with the expression of specific HLA alleles have also been correlated with HIV elite controllers [129–131], although elite control is rarer in children [32]. 

Beyond CTL responses, infant macaques have also been found to produce humoral immune responses with a similar magnitude, breadth, and ADCC capabilities when compared to adult macaques after acute SHIV infection [119]. A comprehensive assessment of immunologic and virologic parameters in infant macaques identified viral load and CD4 + T cell-associated viral RNA in blood (both pre-ART), intact proviral genomes in lymphoid CD4 + T cells, CD4 + T cell-associated viral DNA in rectum, frequency of GranzymeB + CD8 + T cells in lymph nodes, frequency of Ki67 + CD8 + T cells in blood (all pre-ATI), as well as the timing of ART in composite as the best predictors of time to rebound [125]. It remains to be seen whether these parameters can be used as biomarkers in children, and in particular, whether parameters measured in blood (without access to tissues) would be sufficient to inform ATI decisions in cure trials.

## Cure Approaches Tested in Infant Nonhuman Primates

### Broadly Neutralizing Antibodies (bNAbs)

Advances in antibody discovery and cloning techniques have allowed the development of next-generation bNAbs known for their remarkable breadth and potency against HIV. Importantly, bNAbs have antiviral properties beyond neutralization of HIV virions, as in some cases, they can also mediate ADCC [132, 133]. Furthermore, bNAbs in circulation may form immune complexes with viral proteins or virions, enhancing dendritic cell (DC)-mediated antigen presentation and boosting antiviral T cell responses. This immunologic phenomenon has been called the “Vaccinal Effect” of bNAbs [134]. The therapeutic application of bNAbs has been studied in both preclinical and clinical trials [135]. This section will provide an overview of key studies that assessed the efficacy of bNAbs in infant NHPs infected with chimeric SHIVs that encode Env proteins from HIV (Table 1).

**Table 1:** Published studies evaluating cure approaches in infant NHPs

Study	Infection (virus/route)	Age of animals at infection	ART	Cure strategy	Intervention	Highlighted results
Hessell et al. [72]	SHIV_SF162P3_/P.O	1 month	No	bNAbs	PGT121 and VRC07-523 at days 1, 4, 7, and 10 post-infection	• Animals (10/10) that received the bNAb cocktail did not develop viremia for 6 months of follow-up • Four of the animals that did not develop viremia over a period of 6 months underwent CD8 depletion. No viremia was observed • SHIV DNA was observed in all tissues analyzed in control animals (2/2) 14-day post-infection, while no SHIV DNA was detected on the treated animals (2/2)
Shapiro et al. [73]	SHIV_SF162P3_/P.O	1 month	No	bNAbs	PGT121 and VRC07-523 at days 2, 4, 7, and 10 post-infection	• 3/6 animals that received the bNAb combination developed sustained viremia albeit later than unimmunized animals (5 to 6 weeks vs 4 to 7 days, respectively) • The remaining animals (3/6) suppressed viremia except for transient blips • All animals with sustained viremia (3/6) had viral DNA in all tissues analyzed at 39 weeks
Shapiro et al. [73]	SHIV_SF162P3_/P.O	1 month	No	bNAbs	PGT121 and VRC07-523-LS as a single dose at 30-h post-infection	• All animals (6/6) remained aviremic during 24 weeks of follow-up • No viral DNA was detected after necropsy at week 24 in the treated animals • Both bNAbs in this single dose group had significantly longer half-lives when compared to the animals that received multiple doses • The multiple dose regimen was associated with higher levels of ADA^3^
Bricker et al. [136]	SIV_mac251_/P.O	4 to 5 weeks	4-week post infection (TDF/FTC/DTG^2^)	Therapeutic vaccination	2 doses of Ad48/SIV_smE543_ gag-pol-env i.m. 22- and 33-week post-infection 2 doses of MVA/SIV_smE543_ gag-pol-env i.m. 38- and 50-week post-infection 10 doses of TLR7 agonist GS-986 p.o. biweekly from 40- to 60-week post-infection	• The vaccination regimen increased the magnitude and polyfunctionality of cellular responses against SIV_mac239_ • Antibody responses against SIV_mac251_ and SIV_smE543_ were significantly higher than controls 2 weeks after the final immunization • No differences in the size of the reservoir, time to rebound, or the proportion of animals that rebounded post ATI^4^ between vaccinated (*n* = 8) and unvaccinated animals (*n* = 8)
Bricker et al. [137]	SIV_mac251_/P.O	4 to 5 weeks	4-week post infection (TDF/FTC/DTG^2^)	LRA	AZD5582 i.v. once weekly for 10 weeks after receiving ART for > 64 weeks	• A similar proportion of infants (5/8) and adults (5/9) had detectable on-ART viremia induced by AZD5582 • The proportion of viremic events above the limit of detection was lower in infants compared to adults (6% in infants; 46% in adults) • Pharmacokinetic analysis showed lower AZD5582 half-life and peak concentration in infants compared to adults
Deere et al.^1^ [138]	SIV_mac251_/P.O	2 weeks	1-week post infection (TDF/FTC/DTG^2^)	Bispecific antibody	CCR5/CCR3 Bispecific antibodies as a single dose 2 days after ART initiation	• 4/7 animals receiving the bispecific antibodies remained aviremic 2-week post-ATI^4^ • 2/4 aviremic animals at week 2 post-ATI^4^ rebounded at day 97 and 173. The remaining 2 remained aviremic for more than 6 months • One of the aviremic animals for more than 6 months had no evidence of SIV DNA or RNA in the tissues analyzed at necropsy

^1^Preprint

^2^*TDF* tenofovir, *FTC* emtricitabine, *DTG* dolutegravir

^3^Anti-drug antibodies

^4^Analytical treatment interruption

bNAb studies initially focused on the prevention of infection. In newborn rhesus macaques, a combination of neutralizing IgG1 human mAbs targeting gp120 (b12, 2G12) and gp41 (2F5, v4E10) prevented oral infection with SHIV in 50% of animals that received 2 doses of the combination at 1 h and 8-day post-challenge, while those that developed infection had slower disease progression and conserved CD4 + T cell counts [139]. Hessell et al. conducted a study using more potent bNAbs for post-exposure prophylaxis (PEP) following oral SHIV challenge [71]. Administration of VRC07-523 (which targets the CD4 binding site) and PGT121 (which targets a V3-glycan-dependent site) through subcutaneous injections at 1, 4, 7, and 10 days after viral exposure prevented viremia. Furthermore, while SHIV DNA was detected 2 days after challenge in the oral mucosa and the draining oral lymph nodes in both treatment and control animals, by day 14, only the control animals had evidence of viral dissemination. This result was interpreted to indicate that passive immunization with bNAbs soon after exposure can clear infected cells before the latent reservoir (or an immune response) is established.

However, evidence suggests that the window of opportunity to limit reservoir formation with bNAbs is narrow. The same group assessed the effect of delaying the initiation of bNAb treatment following oral SHIV challenge [72]. In contrast to their previous study, giving bNAbs 48 h after challenge (with subsequent doses on days 4, 7, and 10) did not prevent the onset of viremia. Nevertheless, half of the infants demonstrated only temporary spikes of viremia, and those that developed sustained viremia did so at a later timepoint than the untreated control group. The development of antidrug (i.e., anti-bNAb) antibodies (ADA) may explain these results. To assess the effect of ADA induced by multiple doses of bNAbs, a single dose of the bNAb cocktail was given 30 h after oral SHIV challenge. Compared to the multiple-dose group, the single-dose group exhibited lower levels of ADA, and both bNAbs in this group had significantly longer half-lives. Moreover, the single-dose group controlled viremia and restricted reservoir seeding [72].

The appeal of bNAbs as an immune therapeutic derives from their effector functions. Unlike ART, bNAbs can mediate the clearance of infected cells via their constant region (Fc) [140], and while it has been demonstrated that combining bNAbs enhances their neutralization capabilities and reduces the possibility of viral escape [141–143], there have been relatively few studies examining how combinations impact the effector functions of bNAbs. In one such study, Berendam et al. investigated both the effector and neutralization functions of a panel of 18 bNAbs targeting various sites on the HIV Env protein to determine the most effective combination strategies. Of the nine combinations tested, seven exhibited ADCC activity against SHIV.C.C505-infected cells. Moreover, some combinations demonstrated strong antibody-dependent cellular phagocytosis (ADCP), and broad neutralization against other tier 2 clade A, B, C, and D SHIVs [133]. Ultimately, the combination of 3BNC117, PGDM1400, and PGT151 showed the best overall effector and neutralization functions against cross-clade SHIVs [133], and the efficacy of this combination in controlling viral rebound after ATI in infant macaques was evaluated. In the group of SHIV.C.CH505-infected infants that received the bNAb combination prior to ART interruption, a significant delay in the time to viral rebound was observed [144]. Our group observed similar results [144], using a combination of neutralizing mAbs isolated from rhesus macaques targeting different regions of the SIV Env [145, 146]. These and other studies indicate the potential of therapeutic bNAbs to achieve post ART viral control [106, 147]. Whether bNAb effector functions and/or vaccinal effects can contribute to the reduction of viral reservoirs, and how this relates to antigenic load, remains to be elucidated in infant models.

Adeno-associated virus (AAV) gene transfer has the potential to deliver bNAbs for an extended period of time as the transduction of long-lived cells (e.g., skeletal muscle cells) can continue to express transgenes for years after a single dose [148]. This property makes AAV vectors a particularly desirable platform for the delivery of bNAbs that overcomes the limitation of repeated dosing to maintain therapeutic levels. Several groups have demonstrated prolonged expression of AAV-delivered bNAbs in adult macaques and in clinical trials [149–151]. Preliminary results from an ongoing study in infant rhesus macaques have also been reported [144]. The pediatric setting seems particularly suited for this approach, as the more tolerogenic immune system may limit the induction of ADA.

### Bispecific Antibodies

Engineered antibody-like molecules against HIV could potentially be used to enhance clearance of the HIV reservoir. Bi-specific T/NK cell engagers (BiTEs/BiKEs) target regions in HIV and bind T or NK cells, respectively [152, 153]. Based on the evidence that allogeneic hematopoietic stem cell transplant using cells from donors with a mutation in the CCR5 gene that abrogates expression is associated with cure [154], Deere et al. tested bispecific antibodies targeting CCR5/CD3. Neonatal macaques were challenged orally with SIVmac251 at 2 weeks of age, initiated on ART 1 week after infection, and given the bispecific antibody 2 days after ART start [138]. Macaques underwent ATI following 18 weeks of ART, and while all control animals experienced viremia within 2 weeks, only 3/7 animals that received the bispecific CD3/CCR5 antibody were viremic in this time frame. Excitingly, two infant macaques remained aviremic for longer than 6 months, and one had no evidence of SIV RNA or DNA in any of the studied tissues.

### Therapeutic Vaccination

Immunization during treated HIV infection is an appealing strategy to enhance antiviral immune responses, and one that would likely have high uptake given much of the world’s acceptance of childhood vaccinations. There have been two recent studies of therapeutic vaccination in infant macaques that tested different approaches to deliver viral antigens. Our group tested a prime-boost regimen of Ad48/MVA vectors encoding SIV_smE543_ Gag/Pol/Env in infant NHPs orally infected with SIV and treated with ART [136]. This regimen, which was also accompanied by the TLR7 agonist GS-986 as a vaccine adjuvant, was modeled after a study conducted in adult macaques. Similar to what was seen in adults [155], infants developed cellular responses against SIV with a significantly higher magnitude, breadth, and polyfunctionality when compared to controls and pre-vaccination levels, and as expected, GS-986 promoted the activation of CD8 + and CD4 + T cells as well as monocytes. However, unlike the adult macaque study, a decrease in the viral reservoir and delayed viral rebound after ATI were not seen. Yagnik et al. tested a DNA/MVA/protein-based therapeutic vaccination regimen in infant macaques, using DNA and MVA vectors expressing SHIV Gag and Env, and a protein-based SHIV Env vaccine. Here again, reservoir levels prior to ATI were not different between vaccinated animals and controls, and no differences in the time to rebound were observed post-ATI, despite vaccine-induced increases in both cellular and humoral immunity [156].

In combination, both studies show that heterologous therapeutic vaccination regimens are well tolerated and able to enhance adaptive immune responses. Nonetheless, it is apparent that further interventions will be needed for clearance of the viral reservoir in the pediatric setting.

### Latency Reversal Agents (LRAs)

Approaches to stimulate CD4 + T cells to reactivate the latent reservoir have been proposed since the discovery of HIV persistence. Given the toxicities associated with generalized CD4 + T cell activation [157], targeted approaches to reactivate the latent provirus are essential, especially for infants and children. Safety concerns have made this area of pediatric cure research particularly understudied. Our group has explored the use of small molecules targeting the non-canonical NF-kB pathway for latency reversal. Specifically, a mimetic of the second mitochondrial activator of caspases (SMACm), AZD5582, was found to be a powerful LRA in SIV-infected, ART-suppressed adult rhesus macaques [158]. However, when AZD5582 was given to infant macaques, the latency reversal effect was milder [137]. Furthermore, an altered gene expression pattern was observed in CD4 + T cells from treated infants versus adults. Research is ongoing to determine if this difference was related to drug metabolism and/or differential reservoir targeting. In line with these results, there is emerging evidence that latently-infected cells from individuals with perinatal HIV infection may be more resistant to reactivation than those with horizontally-acquired HIV [18], making it crucial to understand the mechanisms and pathways involved in latency reversal in children.

## Cure Approaches of Particular Interest for New Pediatric NHP Studies

Numerous cure strategies have not yet been tested in pediatric humans nor in infant NHPs, and some of these may prove more effective in infants and children and/or should be prioritized due to existing safety data from other conditions. It is important to note, however, that perinatal HIV encompasses a wide age range with a highly dynamic immune system, so attention to factors such as route of infection, timing of ART, time to viral suppression, viral blips, ART non-adherence, and current age are going to be critical to consider when designing clinical studies.

Adoptive cell therapy approaches include transferring antigen-specific T cells expanded ex vivo [159]. In immunocompromised children with CMV viremia, adoptive transfer of HLA-matched ex vivo generated CMV-specific T cells have been associated with an increased period of CMV-free survival and remission [160]. It is unclear if the same benefits could be obtained against the HIV reservoir as latent cells have decreased MHC class I expression. However, this methodology could potentially capitalize on the expansion of vaccine-induced T cell responses given in combination strategies with LRAs.

Chimeric antigen receptor (CAR) T cells, which can recognize antigens independently of MHC presentation [161], are a more promising tool for immunological clearance of latent HIV reservoirs. Initial clinical trials with CAR-T cells targeting HIV did not impact viral control or reservoir size [162, 163]. However, newer generation CAR-T cells, designed for enhanced antigen recognition and resistance to HIV infection, have proven effective in humanized mice [164, 165]. Evaluating this strategy in the infant NHP model would be essential to understanding the impact of the increased regulatory T cells in early life on CAR-T cell efficacy. Safety assessments will also be crucial, given the reported severe adverse events in pediatric patients receiving CAR-T cells for hematological malignancies [166].

Chemotherapeutics that target anti-apoptotic pathways are of particular interest, as HIV exploits these pathways to promote the maintenance of latency [167]. Venetoclax, a BH3 mimetic, binds to Bcl-2, releasing Bcl-2 blocked pro-apoptotic proteins and inducing apoptosis in cancer cells [168]. In a recent study, Venetoclax was able to significantly delay viral rebound compared to controls in HIV-infected humanized mice [169, 170]. Furthermore, Venetoclax decreased total and intact HIV DNA in CD4 + T cells from PLWH ex vivo in a dose-dependent manner. This chemotherapeutic is an intriguing strategy in children as retrospective studies and clinical trials in pediatric cancer have demonstrated its safety in this population [170, 171].

Block and lock approaches aim to silence viral gene expression, leading to irreversible latency that would not result in viral rebound in the absence of ART [172]. Several approaches have been studied to achieve viral gene repression including latency promoting agents (LPAs) [172] and genome editing strategies targeting HIV regulatory genes [173]. Challenges in applying this strategy are the overall size of the reservoir and anatomic sites that may not be reached by LPAs or gene therapy delivery systems. However, early treated infants or adolescents and young adults with perinatal HIV and prolonged viral suppression may be ideal candidates for this cure strategy due to the limited size of their reservoirs.

Given the complexity of the HIV reservoir and the pediatric immune response, a pediatric HIV cure would most likely require combination approaches. Strategies that reactivate the reservoir together with immunotherapies that take advantage of increased viral antigen expression to eliminate infected cells (e.g., “shock and kill”) could lead to a decrease in the size of the reservoir and possibly prolonged periods of ART-free viral control [174]. Infant NHP models allow for the assessment of not only efficacy but also the safety and possible favorable or unfavorable interactions. With adequately sized experiments, NHPs can also be used to test components of combination therapies to elucidate individual contributions to an observed effect. Furthermore, the infant NHP model permits interrogation of anatomic sites that are impossible to assess in children to gain a deeper understanding of the effect candidate interventions have on the size of the total body reservoir [175].

## Conclusion

Despite wide knowledge of strategies that prevent vertical HIV transmission, over 100,000 new pediatric HIV infections were diagnosed in 2022. Moreover, children are also less likely than adults to receive treatment, highlighting the pressing need for a pediatric HIV cure. The unique characteristics of the pediatric immune system have significant implications for HIV infection, disease progression, the cells that comprise the HIV reservoir, and the efficacy of interventions aimed at cure. We have described here how infant NHP models recapitulate the main features of HIV pathogenesis and persistence in children and have also proven invaluable in evaluating the effectiveness of novel cure-directed interventions. Future studies in pediatric NHP models will undoubtedly aid in the pursuit of a cure for pediatric HIV.
